# Risk factors and management strategies for cerebrospinal fluid leakage following lumbar posterior surgery

**DOI:** 10.1186/s12893-021-01442-6

**Published:** 2022-01-29

**Authors:** Jin Tang, Qilin Lu, Ying Li, Congjun Wu, Xugui Li, Xuewen Gan, Wei Xie

**Affiliations:** Hubei 672 Orthopaedics Hospital of Integrated Chinese & Western Medicine, No. 279 Luoyu Road, Hongshan District, Wuhan, 430079 Hubei China

**Keywords:** Lumbar posterior surgery, Cerebrospinal fluid leakage (CSFL), Risk factor, Management strategy, Logistic regression analysis

## Abstract

**Objective:**

To analyze the risk factors of cerebrospinal fluid leakage (CSFL) following lumbar posterior surgery and summarize the related management strategies.

**Methods:**

A retrospective analysis was performed on 3179 patients with CSFL strategies lumbar posterior surgery in our hospital from January 2019 to December 2020. There were 807 cases of lumbar disc hemiation (LDH), 1143 cases of lumbar spinal stenosi (LSS), 1122 cases of lumbar spondylolisthesis(LS), 93 cases of lumbar degenerative scoliosis(LDS),14 cases of lumbar spinal benign tumor (LST). Data of gender, age, body mass index(BMI), duration of disease, diabete, smoking history, preoperative epidural steroid injection, number of surgical levels, surgical methods (total laminar decompression, fenestration decompression), revision surgery, drainage tube removal time, suture removal time, and complications were recorded.

**Results:**

The incidence of 115 cases with cerebrospinal fluid leakage, was 3.6% (115/3179).One-way ANOVA showed that gender, body mass index (BMI), smoking history, combined with type 2 diabetes and surgical method had no significant effect on CSFL (*P* > 0.05). Age, type of disease, duration of disease, preoperative epidural steroid injection, number of surgical levels and revision surgery had effects on CSFL (*P* < 0.05). Multivariate Logistic regression analysis showed that type of disease, preoperative epidural steroid injection, number of surgical levels and revision surgery were significantly affected CSFL (*P* < 0.05).Drainage tube removal time of CSFL patients ranged from 7 to 11 days, with an average of 7.1 ± 0.5 days, drainage tube removal time of patients without CSFL was 1–3 days, with an average of 2.0 ± 0.1 days, and there was a statistical difference between the two groups (P < 0.05).The removal time of CSFL patients was 12–14 days, with an average of 13.1 ± 2.7 days, and the removal time of patients without CSFL was 10–14 days, with an average of 12.9 ± 2.2 days, there was no statistically significant difference between the two groups (*P* > 0.05).

**Conclusion:**

Type of disease, preoperative epidural steroid injection, number of surgical levels and revision surgery were the risk factors for CSFL. Effective prevention were the key to CSFL in lumbar surgery. Once appear, CSFL can also be effectively dealt without obvious adverse reactions after intraoperative effectively repair dural, head down, adequate drainage after operation, the high position, rehydration treatment, and other treatments.

## Introduction

Cerebrospinal fluid leakage (CSFL) caused by dural tears (DTs) is a common complication in spinal surgery, especially lumbar operations [1]. According to literature reports, the incidence of CSFL is ~ 2–20% [2–4], which is related to trauma, intraoperative tumour resection, adhesion of the dural sac to surrounding tissues, iatrogenic injury and other factors [5, 6]. Improper handling of CSFL can lead to pseudocysts, delayed incision healing of the stiff backbone or no healing, infection of the incision, infection of the central nervous system, complications and even death [7, 8]. Therefore, CSFL has also gradually drawn great attention from spine surgeons. Lumbar posterior surgery in patients admitted to our hospital from January 2019 to December 2020 were retrospectively analysed to explore the incidence, related risk factors and management strategies of CSFL complicated by lumbar posterior surgery. The relevant data were summarized and are reported as follows.

## Subjects and methods

### Inclusion criteria

1. Based on the patients' medical history, signs and imaging examinations, they were diagnosed with lumbar disc herniation (LDH), lumbar spinal stenosis (LSS), lumbar spondylolisthesis (LS), lumbar degenerative scoliosis (LDS), or lumbar spinal benign tumour (LST).
2. There were unsatisfactory results after systematic conservative treatment for more than 3 months.
3. There were no obvious surgical contraindications.
4. Posterior lumbar decompression (total lamina decompression or fenestration decompression) was present.
5. Patients and their families had good compliance and were willing to cooperate with the treatment and follow-up visits.

### Exclusion criteria

1. Patients underwent cervical and thoracic surgery at the same time.
2. Lumbar infectious diseases were present.
3. Malignant tumours were present in the lumbar spinal canal.
4. Patients or their families had poor compliance and were unwilling to cooperate with the treatment and follow-up visits.
5. There was a present or past history of mental illness.

### Diagnostic criteria for CSFL

1. Dural injury or CSFL was confirmed during the operation.
2. Postoperative headache, dizziness, and vomiting were related to the patient’s position. The incision had reddish blood or clear fluid exudation.
3. A large amount of reddish bloody fluid or clear fluid was drained from the drainage tube or incision after surgery.
4. Reddish bloody fluid or clear fluid accumulated subcutaneously after the incision.

### General data

A total of 3840 patients who underwent lumbar posterior surgery in our hospital from January 2019 to December 2020 were selected as the research subjects; 661 patients were excluded by exclusion criteria. A total of 3179 patients met the inclusion criteria, including 1606 males and 1573 females; 2058 of these patients (1435 males, 523 females) had a history of smoking. The subjects’ ages ranged from 24 to 80 years (mean: 56.3 ± 12.8 years). The body mass index (BMI) was 14.6–35.9 kg/m^2^ (mean: 21.8 ± 6.2 kg/m^2^). There were 807 cases of LDH, 1143 cases of LSS, 1122 cases of LS, 93 cases of LDS, and 14 cases of LST. There were 1660 cases of total lamina decompression and 1519 cases of window decompression. There were 2515 cases of primary surgery and 664 cases of revision surgery. The follow-up time ranged from 6 to 30 months (mean: 15.7 ± 6.3 months). See Table 1 for details.

**Table 1:** General data

Cases	Gender		Age (years)	Disease type					Surgical methods		Revision surgery	
	Male	Female		LDH	LSS	LS	LDS	LST	Total lamina decompression	Window decompression	Not	Yes
3179	1606	1573	56.3 ± 12.8	807	1143	1122	93	14	1674	1505	2485	694

### Surgical process

All operations were performed under general anaesthesia, in the prone position and via the posterior median approach. The spinous process, bilateral lamina and facet joints were exposed layer by layer. For revision surgery, the upper, lower, medial and lateral boundaries of the dural sac were exposed from the normal anatomical structure to avoid CSFL caused by separation in the surgical scar. If CSFL was found intraoperatively, when the ruptured site of the dural sac could be repaired by suture, waiting for the cerebrospinal fluid to fully flow out and not affect the surgical field of vision. If the cauda equina nerve was herniated from the rupture, the nerve was patiently and carefully stripped and then sutured with a noninjury-free thread until there was no obvious cerebrospinal fluid exudation at the rupture site. When the operation was completed, the leakage was covered with artificial dura or gelatine sponge and tightly sutured layer by layer without any dead cavity.

### Postoperative treatment

1. After the operation, the head was positioned low, the feet were positioned high, and the bed tail was ~ 10 cm high. According to the symptoms of low cranial pressure, the patient could increase or decrease this height by 2–3 cm appropriately.
2. Positive pressure drainage was extended to 7–10 days after the operation. The drainage volume was monitored daily to record the colour and properties of the liquid.
3. Antibiotics (ceftriaxone sodium, etc.) that could cross the blood–brain barrier were used.
4. Fluid supplementation was given daily and monitored regularly with electrolytes.
5. The dressing was changed regularly to keep the wound dry and avoid infection.
6. Six to seven days after the operation, the drainage tube was clamped for 24 h to observe the wound and lower limbs. If the wound was dry and the lower limbs showed no obvious decrease in muscle strength, the drainage tube was removed 24 h later, and the mouth of the drainage tube was ligated with silk thread and closed.

### Observation index

Data on sex, age, BMI, duration of disease, diabetes, smoking history, type of disease, preoperative epidural steroid injection, number of surgical levels, surgical methods (total laminar decompression, fenestration decompression), revision surgery, drainage tube removal time, suture removal time, and complications were recorded.

### Statistical analysis

Measurement data are expressed as the mean ± standard deviation. All data were analysed using SPSS 23.0 software. Count data were compared using the chi-square test. Intergroup differences were compared using the independent sample *t* test. Logistic regression analysis was used to analyse the factors with statistical significance in the univariate analysis. *P* < 0.05 was considered statistically significant, and *P* < 0.01 was deemed highly significant.

## Results

### The incidence of CSFL

There were 115 cases of CSFL, with an incidence of 3.6% (115/3179). Intraoperative rupture of the dural sac was found in 93 cases (79 cases of suture repair). Twenty-two cases of delayed CSFL and no obvious dural injury or CSFL were found during the operation.

### Univariate analysis of risk factors for CSFL

One-way ANOVA showed that sex, BMI, smoking history, type 2 diabetes and surgical method had no significant effect on CSFL (*P* > 0.05). Age, type of disease, duration of disease, preoperative epidural steroid injection, number of surgical levels and revision surgery had effects on CSFL (*P* < 0.05). See Table 2 for details.

**Table 2:** Univariate analysis of risk factors for CSFL

Risk factors		CSFL	Not CSFL	Correlation coefficient	*P*
Gender	Male	64	1547	0.0193	0.277
	Female	51	1517		
Age(years)	≤ 40	11	584	0.0666	0.0009
	40–64	35	1157		
	≥ 65	69	1323		
BMI (kg/m^2^)	< 18.5	33	1103	0.0303	0.2316
	18.5–22.9	39	995		
	≥ 23	43	966		
Smoking history	Yes	71	1987	0.0122	0.4931
	No	44	1077		
Type of disease	LDH	6	801	0.4081	0
	LSS	29	1114		
	LS	37	1085		
	LDS	31	62		
	LST	12	2		
Type 2 diabetes	Yes	58	1385	0.0196	0.2685
	No	57	1679		
Duration of disease (years)	< 3	29	1115	0.0788	0.0001
	3–10	27	981		
	> 10	59	968		
Preoperative epidural hormone injection	Yes	68	1256	0.0687	0.0001
	No	47	1808		
Surgical method	Total laminar decompression	67	1593	0.0234	0.1863
	Fenestration decompression	48	1471		
Number of surgical levels	1	18	1028	0.0785	0.0001
	2–3	42	1059		
	> 4	55	977		
Revision surgery	No	42	2473	0.203	0
	Yes	73	591		

### Multivariate analysis of risk factors for CSFL

Multivariate logistic regression analysis showed that type of disease, preoperative epidural steroid injection, number of surgical levels and revision surgery were significantly affected by CSFL (*P* < 0.05) and that the duration of disease and age of the patients were not significantly affected by CSFL (*P* > 0.05). See Table 3 for details.

**Table 3:** Multivariate analysis of risk factors for CSFL

Risk factors	β	SE	Wald	OR	95% CI for Exp (B)		*P*
					Lower part	Upper part	
Age	− 0.345	0.176	3.830	0.708	0.05	0.501	1.001
Type of disease	1.370	0.138	98.752	3.936	3.004	5.158	0
Duration of disease	0.004	0.156	0.001	1.004	0.739	1.363	0.981
Preoperative epidural hormone injection	0.698	0.246	8.061	2.009	1.241	3.252	0.005
Number of surgical levels	0.342	0.174	3.878	1.408	1.002	1.979	0.049
Revision surgery	1.048	0.198	28.146	2.853	1.937	4.203	0

### Drainage tube removal time and removal time of CSFL

Drainage tube removal time of CSFL patients ranged from 7 to 11 days, with an average of 7.1 ± 0.5 days. Drainage tube removal time of patients without CSFL was 1–3 days, with an average of 2.0 ± 0.1 days, and there was a significant difference between the two groups (P < 0.05). The removal time of CSFL patients was 12–14 days, with an average of 13.1 ± 2.7 days, and the removal time of patients without CSFL was 10–14 days, with an average of 12.9 ± 2.2 days. There was no statistically significant difference between the two groups (*P* > 0.05). See Table 4 for details.

**Table 4:** Extubation time and removal time of CSFL patients

	Extubation time (days)	Removal time (days)
CSFL	7.11 ± 0.48	13.11 ± 2.67
No CSFL	2.02 ± 0.13	12.87 ± 2.19
t	341.93	1.144
*p*	0	0.253

### Postoperative complications of CSFL

There were 29 CSFL patients (25.2%) with low cranial pressure, and the symptoms were gradually relieved after raising of the bed and sufficient fluid infusion. There were 6 patients (5.2%) with lower limb pain and numbness after extubation, which were gradually relieved after local physical therapy, dehydration and detumescence, local suction and other treatment and did not lead to serious nerve injury. No intraspinal infection or intracranial haemorrhage or delayed healing of the incision, death or other complications occurred. There were no long-term complications, such as dural pseudocysts, at the last follow-up.

#### Typical case

**Fig. 1:**
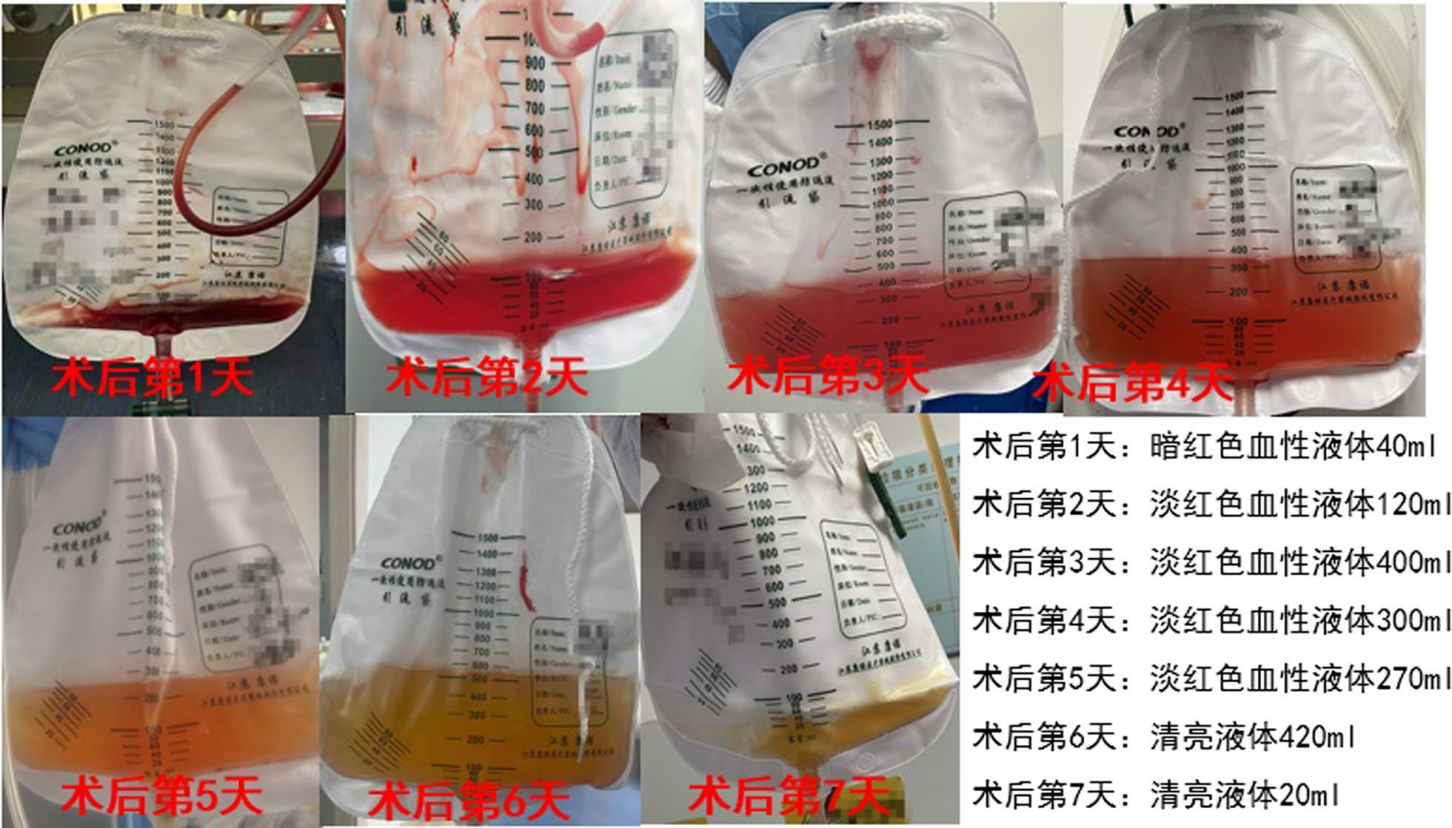
Postoperative drainage was shown in Fig. 1

Patient Song X X, male, 48 years old, was admitted to the hospital due to “low back pain with left lower limb pain and anaesthesia for more than 2 years, aggravating for 3 days”. He underwent “L3/4, L4/5 posterior spinal canal decompression, discectomy, pedicle screw fixation + cage bone graft fusion” under general anaesthesia after examination. No obvious CSFL was found intraoperatively, and 40 ml dark red bloody fluid was drained on the 1st day after the operation, 120 ml reddish bloody fluid was drained on the 2nd day, and 400 ml reddish bloody fluid was drained on the 3rd day, which was considered postoperative delayed CSFL ( Fig. 1).

**Fig. 2:**
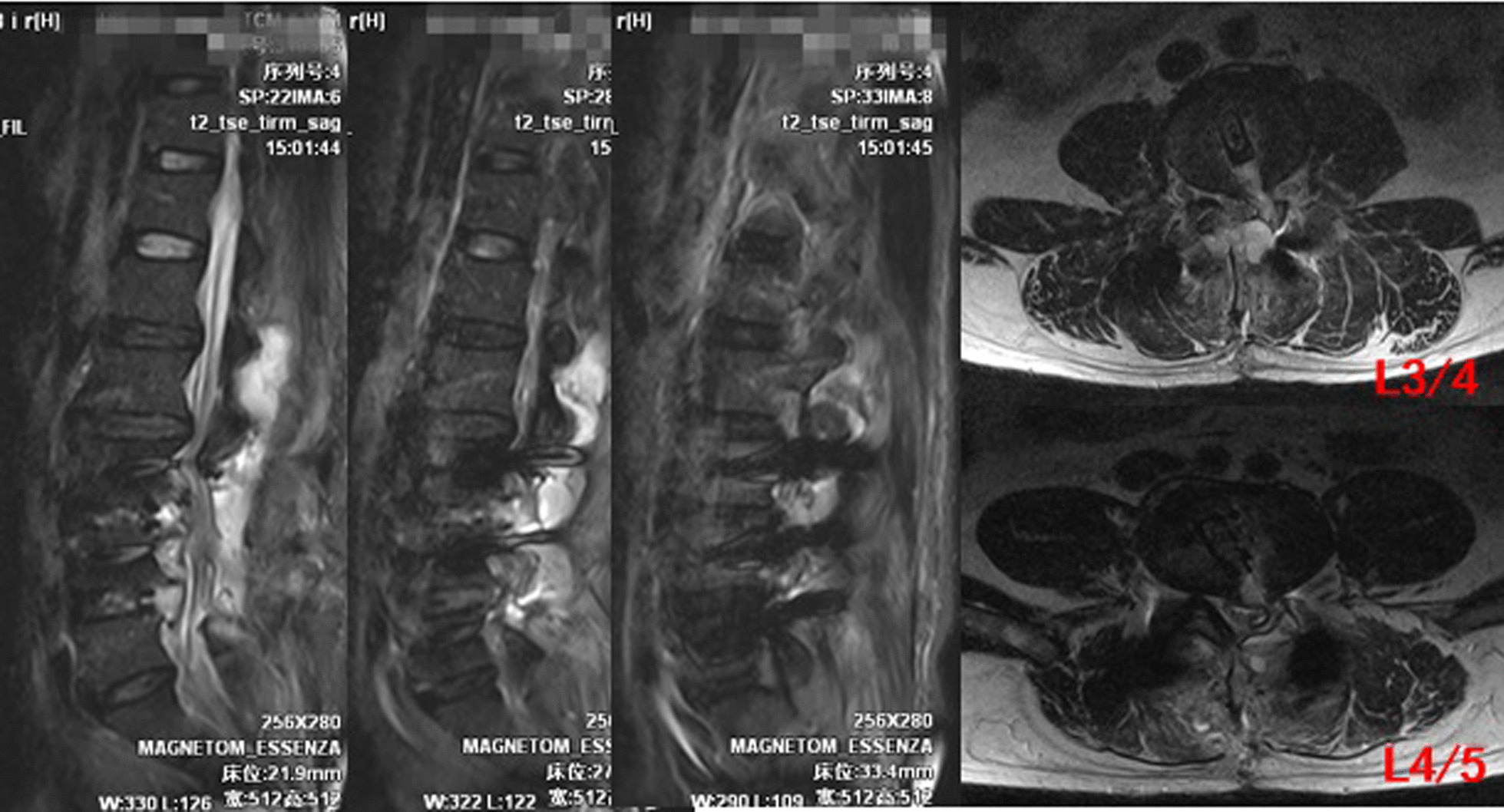
MRI was reexamined on the 12th day after the operation

**Fig. 3:**
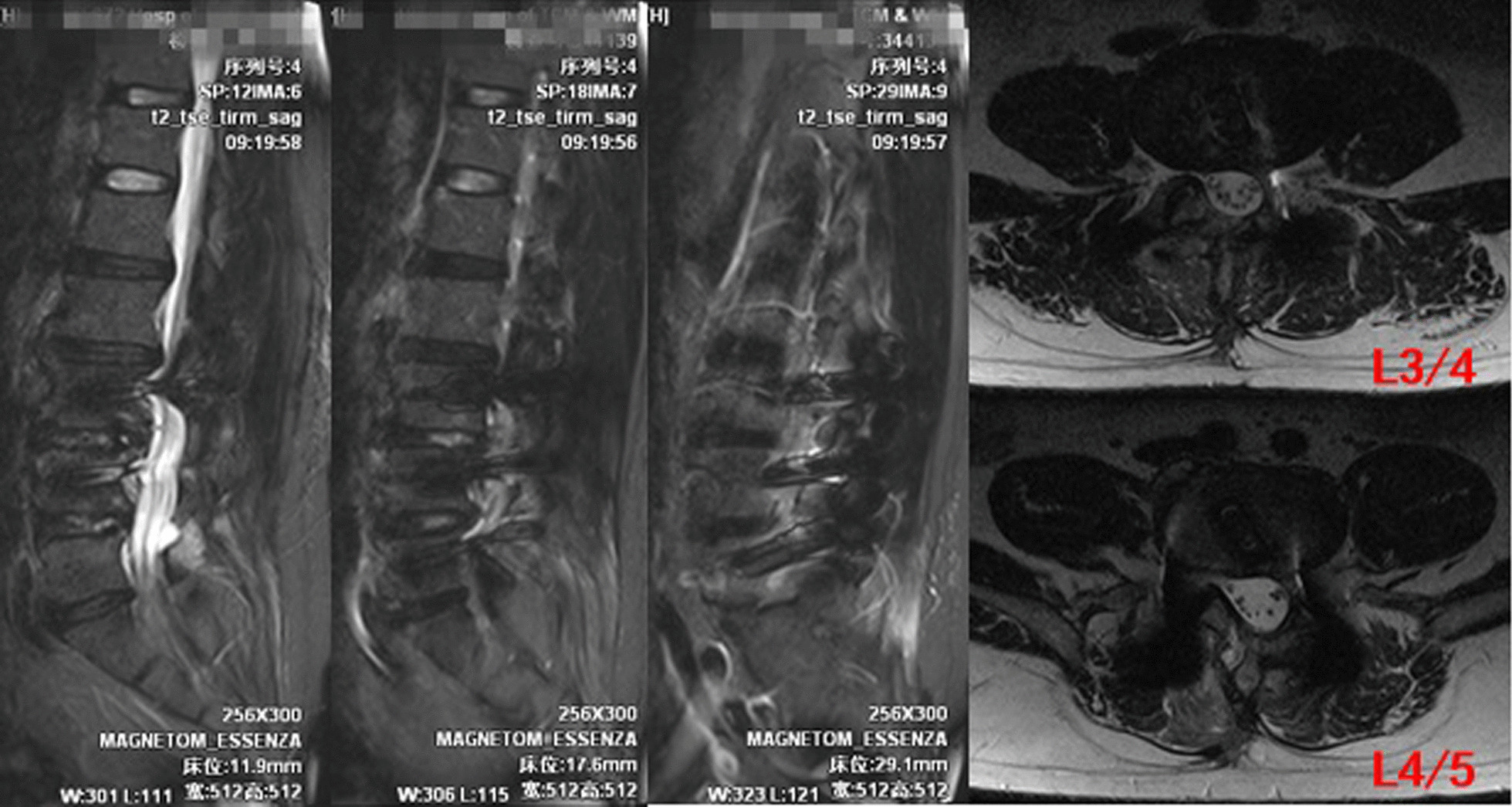
MRI was re-examined more than 3 months after the operation

The drainage tube was removed, and the drainage tube orifice was sutured and closed with one stitch. MRI was re-examined on the 12th day after the operation (see Fig. 2 for details). The wound was sutured and healed on the 13th day after the operation. MRI was re-examined more than 3 months after the operation (see Fig. 3 for details).

## Discussion

### The incidence of CSFL

According to the literature reports, the incidence of CSFL is ~ 2–20% [2–4]. The incidence of CSFL in primary lumbar surgery ranges from 5.5 to 9.0%, while that in revision surgery ranges from 13.2 to 21.0% [9]. Koji et al. [3] retrospectively analysed 2146 patients who underwent lumbar posterior surgery in 8 hospitals, and the overall incidence of CSFL was approximately 7.7% (166/2146), among which the incidence of CSFL was 7.5% (123/1644) for LSS, 38.9% (7/18) for OPLL/OYL, 5.5% (23/422) for LDH, and 21.0% (13/62) for LDS. In this study, the overall incidence of CSFL was 3.6% (115/3179): 0.7% (6/807) for LDH, 2.5% (29/1143) for LSS, 3.3% (37/1122) for LS, 33.3% (31/93) for LDS, and 85.7% (12/14) for LST. The incidence of CSFL in initial surgery was approximately 1.7% (42/2515) and that in revision surgery was ~ 11.0% (73/664), which was slightly lower than that reported in the domestic and foreign literature. This finding might be related to the status of our hospital as a specialized orthopaedic institution. Lumbar surgery is now a routine operation in our hospital, and doctors at all levels have mastered the various operations and formed our own characteristic procedures.

### Possible reasons for CSFL

Possible reasons for CSFL mainly include the following: (1) Trauma: CSFL caused by lumbar burst fracture tearing dura, and CSFL caused by bone fragments protruding towards the spinal canal puncturing the dura [10]. (2) Patients' own factors: long course of disease, severe spinal stenosis, herniated disc tissue, hyperplastic bone block or ligament adhesion to the dural sac [11]. After multiple operations, the dural sac adheres to the surrounding tissues. The wall of a spinal canal tumour is a part of the dural sac, and the partial dural sac is removed when the tumour is completely removed [12]. (3) Iatrogenic factors: Iatrogenic injuries are the main cause of most CSFLs [13] and involve insufficient preoperative preparation, insufficient estimation of intraoperative difficulties, residual sharp bone edge injury of the dura mater, intraoperative injury of the dural sac [14], inexperience of the operator, careless operation, etc. (4) Unexplained CSFL: spontaneous CSFL, which may be related to dural dysplasia and degeneration. In such cases, there is no obvious CSFL in preoperative fracture films or intraoperative dural lesions, but hidden CSFL appears after the operation.

### Risk factors for CSFL

Multivariate regression analysis showed that the risk factors for CSFL were type of disease, preoperative intraspinal hormone injection, number of surgical levels and revision surgery (*P* < 0. 05). Intradural tumours, degenerative scoliosis and severe LSS have been previously reported as high-risk factors for CSFL [3, 13]. In our study, the OR of the type of disease was 3.9, with a 95% confidence interval of 3.0–5.2, *P* < 0.01, indicating that the type of disease had a significant effect on patients with CSFL. The incidence of CSFL was 85.7%, that of degenerative scoliosis was 33.3%, that of spondylolisthesis was 3.3%, that of LSS was 2.5%, and that of lumbar disc herniation was 0.7%.

Studies [15] have shown that a long course of disease and long-term dural pressure lead to a reduction in epidural fat, thinning of the dural thickness, and expansion of the dura after laminectomy and that DTs are prone to occur during decompression. However, in our multivariate study, the OR of disease duration was 1.0, and the 95% confidence interval was 0.7–1.4, *P* > 0.05, indicating that duration of disease was not a risk factor for CSFL.

In our study, the OR of preoperative epidural steroid injection was 2.0, with a 95% confidence interval of 1.2–3.3, *P* < 0.01, indicating that preoperative epidural steroid injection was also one of the risk factors for CSFL. We considered that the hormones injected into the spinal canal (triamcinolone acetonide, etc.) were mostly macromolecules, which could not be completely absorbed by the body. They accumulated around the lesions in the spinal canal and formed adhesions between the dural sac. In the process of decompression, dural rupture easily formed, leading to CSFL.

The number of surgical levels was also one of the risk factors for CSFL. In our study, the incidence of CSFL was 1.7% (18/1046) in patients with 1 level of decompression, 3.8% (42/101) in patients with 2–3 levels of decompression, and 5.3% (55/1032) in patients with more than 4 levels of decompression. With the increase in the number of surgical levels, the risk of CSFL also increased, which was basically consistent with the results of foreign studies [16]. A possible reason may be that with the increase in the number of surgical levels, more extensive treatment is needed. The incidence of CSFL was expected to be higher when the dural sac was exposed for a longer time. Meanwhile, the operative time was longer, the energy consumption of the operator was greater, and the operation was not meticulous, which increased the chance of iatrogenic dural injury.

The incidence of CSFL in revision surgery was 11.0%, which was 2.9 times higher than that in primary surgery, suggesting that this factor had a greater impact on the occurrence of CSFL. In revision surgery, the vertebral lamina and other bone structures in the surgical area had been removed, the anatomical structure was different from normal, the dura mater was widely adhered to the surrounding scar tissue, and dura mater injury easily occurred in the process of surgical operation to release the nerve root and dura mater [16, 17].

### Management of CSFL

Combining our experience and the related literature, we summarize the following management methods:

1. Intraoperative management
  1. Once CSFL occurred, according to the degree of dural injury, the dura was immediately, patiently and carefully repaired. Early and timely detection of CSFL and tight suturing of the dura mater were the main methods to prevent postoperative CSFL [18]. In our study, 93 cases of dural sac rupture were found during the operation, of which 79 underwent suture repair.
  2. Artificial spinal patch repair, subcutaneous fascia or deep muscle fascia coverage, and fibrin glue sealing can be considered the choice of dural rupture repair during surgery [5]. In our study, all patients with CSF were covered with an artificial dura mater or deep fascia layer.
  3. Close suturing of muscle, fascia and skin, especially the deep fascia layer, plays a beneficial role in preventing postoperative CSFL [19]. Tight suturing of deep fascia was the key to ensuring good wound healing in patients with CSFL.
2. Postoperative management
  1. Bed rest. Keeping a reasonable position and using head low and feet high (raising the bed tail approximately 10-15 cm) could prevent symptoms of low intracranial pressure and reduce the pressure of CSF on dural breaks, which was conducive to their healing.
  2. Choosing antibiotics that can pass through the blood–brain barrier to prevent the occurrence of intracranial infection and appropriately prolong the use time of antibiotics.
  3. Frequently changing dressings, keeping the wound dry, strengthening nursing, hydrating the intestines and promoting defecation, and reducing cough can promote an increase in CSF pressure caused by abdominal pressure, which is conducive to the healing of the dura.
  4. Strengthening fluid supplementation, maintaining the balance of water and electrolytes, and properly supplementing protein can promote dural healing.
  5. Prolonging the time of drainage tube placement. In our study, the drainage tube time of the CSFL patients was 7-11 days (average: 7.1 ± 0.5 days). The drainage tube was clamped for approximately 7 days to observe the wound and muscle strength of both lower limbs for 24 hours. If the wound was dry and there was no obvious motor sensory disturbance of either lower limb, the drainage tube could be removed. If there was exudation on the wound surface and obvious motor sensory disturbance of both lower limbs, the drainage tube was opened in time to continue drainage for 1–2 days. The time of extubation was judged by the above steps again. In our study, the drainage tube was removed from 3 patients for 11 days. If the drainage tube could not be removed, another operation should be performed to repair the dura. In our study, 1 patient underwent surgery again, and a dural break was found during the operation. No CSFL occurred after dural repair.

### Complications of CSFL

CSFL causes the loss of CSF, which reduces intracranial pressure and leads to postural headache, dizziness, nausea, vomiting and other symptoms of low intracranial pressure [20]. In our study, 29 patients with low intracranial pressure symptoms were gradually relieved after raising of the bed and full fluid supplementation. If CSF accumulates in the incision and compresses the related nerves, this leads to lower limb pain, numbness and even paralysis [21]. In our study, 6 patients with lower limb pain and numbness symptoms were treated with local physical therapy, dehydration and detumescence, and local suction, and the symptoms were gradually relieved without causing serious nerve injury. CSFL could lead to an incision that is directly exposed to the outside environment, which easily causes incisional infection. If pathogenic microorganisms are retrograded with CSF, they can cause spinal canal and intracranial infection, endangering the life of patients [22]. In the present study, there were no complications, such as intraspinal infection, intracranial haemorrhage or delayed healing of the incision. At the end of the follow-up period, there were no long-term complications, such as dural pseudocysts.

## Conclusion

CSFL was found to be common in lumbar posterior surgery. Type of disease, preoperative epidural steroid injection, number of surgical levels and revision surgery were risk factors for CSFL. Although various methods were taken to prevent CSFL, there were no absolute means to prevent it. Effective prevention was the key to avoiding CSFL in lumbar surgery. Once it appears, CSFL can also be effectively managed without obvious adverse reactions after effective intraoperative repair of the dura, downward positioning of the head, adequate drainage after the operation, high positioning of the feet, rehydration treatment, and other therapies. However, due to the limitations of this study, such as its retrospective design and selection bias, the results may be biased and need to be further confirmed by multicentre prospective studies.

## Data Availability

The datasets used and/or analyzed during the current study are available from the corresponding author on reasonable request.

## Abbreviations

BMI: Body mass index
CSFL: Cerebrospinal fluid leakage
DT: Dural tears
LDH: Lumbar disc hemiation
LDS: Lumbar degenerative scoliosis
LS: Lumbar spondylolisthesis
LSS: Lumbar spinal stenosi
LST: Lumbar spinal benign tumor
